# Polyphenol Polymerization by an Alternative Oxidative Microbial Enzyme and Characterization of the Biological Activity of Oligomers

**DOI:** 10.1155/2018/3828627

**Published:** 2018-04-23

**Authors:** Patrizia Di Gennaro, Valentina Sabatini, Silvia Fallarini, Roberto Pagliarin, Guido Sello

**Affiliations:** ^1^Department of Biotechnology and Biosciences, University of Milano-Bicocca, Piazza della Scienza 2, 20126 Milan, Italy; ^2^CRC Materiali Polimerici (LaMPo), Department of Chemistry, University of Milan, Via Golgi 19, 20133 Milan, Italy; ^3^Department of Chemistry, University of Milan, Via Golgi 19, 20133 Milan, Italy; ^4^Department of Pharmacy, University of Piemonte Orientale, Largo Donegani 2, 28100 Novara, Italy

## Abstract

The recombinant catalase-peroxidase HPI from* E. coli* was used as an alternative enzyme in polymerization reactions for the production of (−) epicatechin oligomers and their biological activity was characterized. The enzyme was prepared in two forms: a purified and an immobilized form. Both were tested for their activity in oxidative polymerization reactions, and their stability and reusability were assessed. The polymerization reactions were followed by SEC-HPLC analyses, and the substrate was completely converted into one or more polymerization products depending on the reactions conditions. Results showed that the utilized conditions allowed for the isolation of some oligomers of different molecular weight: the oligomers containing 6 and 7 units of epicatechin substrate are the heaviest ones. Epicatechin was also used in reactions catalyzed by HRP in the same reaction conditions for comparison. In addition, one selected oligomer obtained by HPI enzyme catalysis was shown to act as* in vitro* inhibitor of tumor cell growth, like one oligomer deriving from epicatechin by HRP catalysis. These data confirm that epicatechin oligomeric form is more effective than its monomer in biological activity and suggest the use of HPI as an alternative enzyme in reactions for the production of epicatechin oligomers.

## 1. Introduction

Phenol polymers are interesting compounds in various applications and their preparation has been pursued since many years [1–4]. More recently, the possibility of having a green alternative to classical catalysis has increased the amount of enzymes utilized in this field. Two different enzyme polymerization mechanisms emerged: the hydrolytic and the oxidative mechanisms. Among oxidative catalysts a special position is covered by plant and fungal enzymes, HRP (Horse Radish Peroxidase), and laccases representing the most used catalysts [5].

Peroxidases perform a broad range of radical-mediated polymerization reactions [5–7]. The reactions take advantage of the ability of peroxidases to catalyze the formation of free radicals that, in the appropriate conditions, undergo spontaneous polymerization [8]. Enzymatic polymerization of phenolic compounds performed by peroxidases mainly from fungal and plant origin has been deeply studied [5, 9–11]. The advantages of phenolic compound polymerization by these enzymes are as follows: the possibility of controlling the polymer structure to modify its characteristics and its solubility by changing the reactant ratio, solvents, and temperature and the easy isolation and purification procedures of the obtained compounds [7, 12–14].

Bacterial peroxidases are much less studied and used. Nevertheless, they can represent another source of enzyme-based catalysts. Among these, the catalase-peroxidase HPI from* Escherichia coli *[15, 16] is one of the most known members of the class I catalase-peroxidase. These enzymes transform the substances containing the peroxide bond, preferentially hydrogen peroxide to oxygen and water [17–19]. In addition to their catalase activities (with *V*max values in the order of 2,000–6,000 U/mg) [20], these enzymes function as broad specificity peroxidases, oxidizing various electron donors, including ABTS and* o*-dianisidine [5, 21, 22]. HPI is an efficient peroxidase with an activity on* o*-dianisidine of 8.3 U/mg at pH 4.25 [16, 20]. In a recent paper, its use as a polymerization catalyst has been reported [16].

Polyphenols are an important subset of phenols. Most of them are of natural origin and their functions are mainly related to organism protection through their antioxidant power [1–3, 23]. The use of biocatalysis is considered a powerful alternative for their polymerization and HRP and laccases have their intensive use [5, 6]. However, in our knowledge this is the first time that the use of bacterial peroxidases like HPI protein for the polymerization of polyphenol compounds is described. For this, the use of a very convenient and easy-to-prepare bacterial peroxidase as HPI in order to make polyphenol polymers could add an important point to its exploitation as a multifaceted catalyst.

Polyphenols have been used in veterinary and human applications. These compounds are known for their broad range of biological activity; they act as anticancer, anti-inflammatory, antiallergic, and antiviral agents. In particular, catechins are the most studied compounds also for their ability to regulate the cycle in human cell lines [24–28].

It is also known that oligomers derived from polyphenols are sometimes more effective than the corresponding monomers; therefore, polyphenol polymerization has become a matter of study.

Some preliminary data from Nagarajan et al. suggest that epicatechin polymers obtained by HRP polymerization catalysis could be more effective than the corresponding monomer in tumor cell line growth inhibition [4]. It is thus straightforward to choose epicatechin as the substrate in our experiments to test if the obtained products show a comparable activity.

In this paper the results of polymerization reactions catalyzed by HPI enzyme to obtain polymers of low molecular weight from epicatechin are presented. In addition, it is shown that the obtained oligomers can act,* in vitro,* as inhibitors of tumor cell growth like oligomers derived from epicatechin by HRP peroxidase catalysis.

## 2. Materials and Methods

### 2.1. Crude Extract Preparation

*E. coli* M15[pREP4] (pQE30-KATG) strain was grown at 37°C in 500 mL of LB medium supplemented with ampicillin and kanamycin and 1 mM IPTG as reported by Di Gennaro et al. [16]. After 6 h from induction at 30°C, cells were harvested by 10 min centrifugation at 6,000 rpm.

A pellet of cells equal to OD_600_ = 80 was resuspended in 10 mM phosphate buffer pH 7 and treated with 10 mg/mL of lysozyme for 30 min at 4°C. Then cells were disrupted by sonication (five cycles of 10 s). The cell lysate was centrifuged for 30 min at 12,000 rpm at 4°C, and the clear supernatant (soluble fraction) was analysed by Bradford method as reported in Di Gennaro et al. [16].

### 2.2. Enzyme Immobilization

Crude extracts (120 mg) containing the recombinant protein were added to 4 mL of equilibrated Co^2+^ affinity resin (Talon®, Clontech) and incubated for 20 min at 4°C. The resin was stored in the equilibration buffer (Na_3_PO_4_ 50 mM, NaCl 300 mM, pH 8) at 4°C.

### 2.3. His-HPI Protein Purification

The His-HPI protein purification was performed with the procedure described by Di Gennaro et al. [16]. The protein concentration of eluted aliquots was determined by Bradford method and a sample of each of the eluted fractions was analysed by electrophoresis on a 10% SDS-polyacrylamide gel (Novex Bis-Tris Gels 10%, Invitrogen) and visualized with Comassie Brillant Blue to verify the elution of protein [16]. Imidazole was removed by a membrane dialysis tube with a 12,000-Da cut-off against 10 mM phosphate buffer pH 7 overnight at 4°C.

### 2.4. Peroxidase Activity Assay of HPI

To assess the peroxidase activity of HPI in all its forms we used a standard procedure. Protein sample (containing 0.02 mg/mL of crude extract or 0.0002 mg/mL of purified protein) was added to a solution containing 1.0 mM pyrogallol in 10 mM phosphate buffer pH 7.0 and 0.88 mM H_2_O_2_ [15]. Data were normalized using as control a solution containing 1.0 mM pyrogallol in the appropriate phosphate buffer and 0.88 mM H_2_O_2_ without protein addition. All the reactions were carried out at 30°C for 4–10 min, and all optical spectra were recorded with a Varian Cary 3 UV-visible spectrophotometer. The molar extinction coefficient was 2640 M^−1^ cm^−1^ at 430 nm. The specific activity was determined as *μ*mol × mg^−1^  × min^−1^.

### 2.5. Stability of the His-HPI in Different Conditions

All forms of HPI (HPI immobilized to resin, HPI purified enzyme) were tested for time stability measuring peroxidase activity on pyrogallol. Different storage conditions of the 6x His-HPI protein were used. In particular, different times of storage at 4 and −20°C were used for each form. The activity was determined as reported above.

### 2.6. Polymerization Reactions

Polymerization reactions were carried out as reported by Di Gennaro et al. [16]. The reactions were performed in a final volume of 2 mL for test reactions and 10 mL for preparative reactions with 0.1 mg/mL of purified HPI protein and 1 mg/mL of substrate. Polymerization reactions were performed also with HRP enzyme (Sigma-Aldrich, 150–250 U/mg, salt-free) in the same conditions utilized with HPI. The reactions were initiated by the addition of H_2_O_2_. When the substrate concentration remained constant by HPLC analysis, the reactions were stopped and analysed.

The consumption of the substrate was monitored at 280 nm by HPLC-RP chromatography, using a reverse phase LC-18 column with H_2_O/CH_3_CN 80 : 20 at 1 mL/min flow.

In order to perform the analysis of the polymerization products they were isolated. Depending on the reaction products we used a stepwise procedure. In presence of a solid precipitate the suspension was centrifuged (6000 rpm, at 4°C), the supernatant separated, and the solid washed twice with water. The solid was then overnight dried in an oven at 70°C. The supernatant, as all the solutions directly coming from the reactions, was acidified to pH 3 and extracted three times using a mixture of butanol : ethyl acetate 1 : 9. The merged organic phases were dried on Na_2_SO_4_ and filtered, and the solvent evaporated at retention time using a gentle N_2_ flow.

### 2.7. Polymerization Product Characterizations

#### 2.7.1. First Size-Exclusion Analysis (SEC-1)

This system has been used to determine the molecular weight (MW) of some samples. The MW has been determined using a SEC system based on a Waters 1515 Isocratic pump, 4 Phenomenex columns (Phenogel 5 × 10^−3^ Å-5 × 10^−4^ Å-5 × 10^−5^ Å-500 Å connected together), and an RI detector or an UV detector. The analyses have been performed at r.t., at 1 mL/min solvent (THF) flow and 40 *μ*L of sample. Samples were prepared dissolving 1 mg of solid sample in 200 *μ*L of THF (HPLC grade). MWs are expressed in polystyrene (PS) equivalents. Calibration has been realized using PS standards, with the following nominal peak molecular weight (MP) and molecular weight distribution (*D*) values: Mp = 1600000 Da (*D* ≤ 1.13), Mp = 1150000 Da (*D* ≤ 1.09), Mp = 900000 Da (*D* ≤ 1.06), Mp = 400000 Da (*D* ≤ 1.06), Mp = 200000 Da (*D* ≤ 1.05), Mp = 90000 Da (*D* ≤ 1.04), Mp = 50400 Da (*D* = 1.03), Mp = 30000 Da (*D* = 1.06), Mp = 17800 Da (*D* = 1.03), Mp = 9730 Da (*D* = 1.03), Mp = 5460 Da (*D* = 1.03), Mp = 2032 Da (*D* = 1.06), Mp = 1241 Da (*D* = 1.07), Mp = 906 Da (*D* = 1.12), and Mp = 478 Da (*D* = 1.22), with ethyl benzene (PM = 106 g/mol). 1,2-Dichlorobenzene has been used as internal standard.

#### 2.7.2. Second Size-Exclusion Analysis (SEC-2)

This system has been used to calculate the MWs of the remaining products. The MW has been determined using a SEC system based on a Waters 600E pump, a Waters 486 UV detector, and a Phenomenex column (Phenogel 5 *μ*, 100 Å). The analyses have been performed at r.t., at 0.3 mL/min solvent (DMF) flow and 20 *μ*L of sample. Samples were prepared dissolving 1 mg of solid in 1 mL of DMF (HPLC grade). The MWs have been calculated from a correlation line prepared using the retention times and MWs of three standards: epicatechin, oligomer at 1588 mu, and oligomer at 2038 mu. All measures were repeated three times.

#### 2.7.3. MALDI-TOF Analyses

MALDI-TOF analyses were performed using the instrument MALDI-TOF MICROFLEX, Bruker Daltonics. DHB (gentisic acid) has been used as the matrix. Samples were dissolved in MeOH. Two series of peaks have been obtained: 2038-1750-1462-1174-886 and 1570-1282-994-706.

### 2.8. Biological Activity Tests

#### 2.8.1. Preparation of Cell Line Cultures

The human A375 and WM 266-4 melanoma cell lines were maintained in DMEM, supplemented with 10% fetal bovine serum (FBS), 2 mM L-glutamine, 100 U/mL penicillin, and 100 mg/mL streptomycin (GE healthcare, Milan, Italy). Twice a week, cells were detached with trypsin/EDTA (GE healthcare), counted, and reseeded in a fresh culture medium at different densities.

#### 2.8.2. Cell Proliferation Determination

Cell proliferation was measured by the 3-(4,5-dimethylthiazol-2-yl)-2,5-diphenyl-tetrazolium bromide (MTT) assay [23]. A375 or WM 266-4 cells were seeded (0.5 × 10^5^ cells/well) in 24-well plates and treated with increasing concentrations (0.01–10 *μ*g/mL) of each compound for 3 or 6 days at 37°C in a 5% CO_2_ humidified incubator. The percentage of cell proliferation was calculated as [100(*x* − *y*)/(*z* − *y*)], where *x*, *y*, and *z* were the absorbance read in compound-treated, resting, and compound-untreated cells, respectively.

#### 2.8.3. Cell Cycle Analyses

The cell cycle was analysed by measurement of the cellular DNA content using the fluorescent nucleic acid dye propidium iodide (PI) to identify the proportion of cells that are in each stage of the cell cycle. The assay was carried out using flow cytometry with a fluorescence-activated cell sorter (FACS). A375 or WM266-4 cells were plated in p35 dish at a density of 5 × 10^5^ cells/dish in serum-free medium. After 24 h of incubation at 37°C, cultures were changed to complete medium and 10 *μ*g/mL of each of the compounds was added to the cells. After 3 or 6 days of incubation, cells were trypsinized, pelleted by centrifugation (400*g* for 5 min), fixed by 70% ethanol, and stored at −20°C O/N. Fixed cells were stained in Tris-buffered saline (TBS) containing 50 *μ*g/mL PI and 10 *μ*g/mL RNase free of DNase. They were incubated in the dark for 1 h at 4°C. Cell cycle analysis was performed by FACS (FACSVantage-SE® flow cytometer, BectonDickinson, Milan, Italy) at 488 nm.

#### 2.8.4. Cell Viability

A375 or WM266-4 cells were labelled with 1 mM Calcein-AM (CAM) (Molecular Probes, Invitrogen) in serum-free PBS for 15 min at 37°C in the dark. After being washed, labelled cells were seeded in 24-well plates and allowed to adhere overnight at 37°C in a humidified incubator. Then, cells were treated with 10 *μ*g/mL of each of the compounds for 6 days at 37°C in a 5% CO_2_ humidified incubator. After incubation the cells of each well were harvested, washed, and labelled with propidium iodide (PI), and the viability was measured by flow cytometry. Live cells were identified as CAMhigh/PI− population, apoptotic cells were CAMlow, and late apoptotic/necrotic cells were CAMlow/PI+. The percentage of live, apoptotic, and late apoptotic/necrotic cells were calculated by FACSDiva software and expressed as the percentage of CAMhigh/PI−, CAMlow, and CAMlow/PI+ population relative to untreated cells.

### 2.9. Statistical Analysis

Results were expressed as means ± SEM of at least three experiments. Statistical significance was evaluated by the one-way ANOVA followed by Student's *t*-test for paired populations, using GraphPad Prism 4 (GraphPad Software, Inc., San Diego, CA, USA). Differences among means were considered significant when *p* ≤ 0.05.

## 3. Results

### 3.1. 6x His-HPI Preparation from* E. coli* M15[pREP4] (pQE30-KATG) Crude Extract

To purify the 6x His-HPI recombinant protein, the* E. coli* M15[pREP4] (pQE30-KATG) strain was used. The crude extract was prepared and purified as described in a previous paper by Di Gennaro et al. [16]. The fractions were stored at −20°C. To prepare the HPI enzyme immobilized to resin, the enzyme loaded resin was stored in the equilibration buffer; samples were stored at 4°C and were added to reaction solutions when needed.

### 3.2. HPI Peroxidase Activity

**Figure 1:**
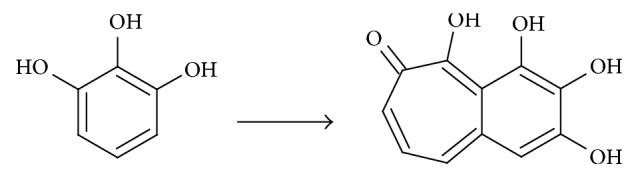
Dimerization of pyrogallol catalyzed by peroxidases.

HPI activity is usually measured using* o*-dianisidine as the reference substrate [15]; however, in our experience* o*-dianisidine cannot be used to measure the activity of the immobilized HPI because it is irreversibly adsorbed by the resin. In order to have a common substrate to measure and compare enzyme activity in all its forms, pyrogallol was selected as the reference substrate. Pyrogallol can be oxidized to purpurogallin by peroxidases (Figure 1). The HPI activity on pyrogallol was measured in the presence of the enzymatic crude extract and in the presence of both the enzyme immobilized on IMAC-Co^2+^ resin and the purified enzyme. The crude extract showed an activity on pyrogallol of 2.5 × 10^−1^ *μ*mol × mg^−1^  × min^−1^. The activity of the crude extract when conserved at 4°C significantly decreases after 28 days to 3.0 × 10^−2^ *μ*mol × mg^−1^  × min^−1^; in contrast, when conserved at −20°C it remains more or less constant over two months. This permits the use of the extract without needing a fresh preparation.

In order to have the biocatalyst in a form easy to manage and recycle, the same resin used for its purification (IMAC Co^2+^) was used for its immobilization. Although this operation is fast and selective, it was necessary to test both the enzyme activity and the stability of the enzyme in this form. So, we performed preliminary activity tests on the immobilized HPI form using pyrogallol as the reference substrate in different experimental conditions. Initially, the conditions used to perform the enzyme immobilization were phosphate buffer 50 mM at pH 8 with NaCl 1.8%; but at pH 8 pyrogallol spontaneously dimerizes and therefore a different set of conditions was selected. The results showed that pH 6.5 and NaCl at 0.3% are the best conditions for the activity of immobilized HPI on pyrogallol (4.5 × 10^−1^ *μ*mol × mg^−1^  × min^−1^); in these conditions the pure resin is inactive. Then, activity retention tests of the enzyme in this form were performed during the time. The enzyme immobilized onto the resin (enzymatic resin hereafter) cannot be frozen because the resin itself does not maintain its characteristics. Therefore, the tests were performed using enzymatic resin conserved at 4°C in the equilibration buffer 50 mM phosphate buffer at pH 8 and with 1.8% NaCl; reactions were performed in the reference conditions. Results showed that the enzyme preserves its activity even after two months (Table 1; value variations are ascribed to the approximation in the resin amount used in each experiment series).

The specific activity referred to pyrogallol oxidation of the immobilized enzyme (0.45 *μ*mol × mg^−1^  × min^−1^) is nearly twice as high as that of the enzyme in the crude extract (0.23 *μ*mol × mg^−1^  × min^−1^). In a parallel series of tests the activity and the activity retention of the purified enzyme were checked. The specific activity of the purified HPI (0.17 *μ*mol × mg^−1^  × min^−1^) is 2.5 times lower than that of the enzymatic resin. In addition, HPI activity is halved after one-month conservation in the equilibration buffer both at 4°C and at −20°C. This result showed that the immobilized HPI is much more stable than the purified enzyme in similar conditions and that it can be stored for more than 2 months at 4°C without loss of activity. Furthermore, the resin form can be easily used several times showing similar efficiency (data not shown).

### 3.3. Epicatechin Polymerization Reactions

Catechins are the principal structural units in condensed tannins. They belong to the group of the 3-flavanols and are present in many varieties of vegetables, herbs, and tees. They are active as preventive of cancer and of inflammatory and cardiovascular diseases, because of their antioxidant action that can limit and remove free radicals [29]. It is also known that in the form of oligomers their activity can be enhanced [4]. To this end, the possibility of substituting the commonly used HRP (Horse Radish Peroxidase) of vegetal origin with the microbial HPI in polymerization reactions was investigated. The polymerization of a catechin as the (−) epicatechin was initially performed in different reaction conditions using HRP as reference. The reaction was followed by the consumption of the substrate that was monitored by HPLC at 280 nm. In all the tested conditions the amount of consumed (−) epicatechin was over the 90% up to a concentration of 3.44 mM in the reaction.

Then, epicatechin polymerization was performed in the same conditions utilized for HRP using the recombinant enzyme HPI (from* E. coli* M15(pREP4) (pQE30-katG)), immobilized onto the IMAC resin. Also, in this case, the decrease of the substrate was monitored by HPLC. The substrate was always completely consumed, except when using a high concentration of epicatechin (17.2 mM) (Table 2). Finally, experiments have been performed using the purified free enzyme HPI. As it is shown in Table 3, the substrate was only partially consumed (60%) even in the presence of a high concentration of protein (0.12 mg/mL; i.e., 12 times the amount present in the experiments with enzymatic resin). Results indicated that HPI can be considered a good biocatalyst in the epicatechin polymerization only in the immobilized form.

### 3.4. Product Analysis and Characterization

To perform the analysis of the polymerization products their preliminary isolation was studied. The formed oligomers are differently soluble in water and they partially precipitate. Because during filtration the oligomers are easily adsorbed on the filters, a procedure that contemplates two steps was used: first, the separation of the solid by centrifugation and, second, the extraction of the water phase with an organic mixture (butanol : ethyl acetate). In addition, the solvent evaporation should be made with care, to preserve the integrity of the products.

The identification of the epicatechin oligomers produced by the biocatalysts required the following procedure. One product was characterized using Maldi mass analysis. Two compounds were then analysed using a first SEC chromatography system; this analysis confirmed Maldi result through system calibration and gave a second product weight. Finally, all the products were analysed using a second SEC chromatography system; using retention times of this analysis and the MWs calculated using the previous analyses a correlation line between retention times and molecular weights was prepared.

Maldi mass analysis clearly shows two sets of peaks. The first set has its highest MW at 2038 mass units, followed by a regular series of peaks each separated by 288 mass units (epicatechin monomer); the second set has its highest MW at 1570 mass units, also followed by a regular series of peaks each separated by 288 mass units. The first series corresponds to a 7-unit oligomer; the second series corresponds to a 6-unit oligomer that lost a group weighting 180 units. This last weight corresponds to the loss of the bicyclic part of epicatechin. At this analysis level it cannot be stated if the second series derives from a self-standing oligomer or from the mass analysis evolution.

In the first SEC analysis the compound MW was estimated using a polystyrene (PS) based calibration; this permits the calculation of an MW of 2326 for the compound used in the Maldi analysis and of an MW of 1724 for a second compound, selected for its well defined difference in chromatography retention time. Using these two MWs and epicatechin as reference, a correlation line between retention time and MW was prepared using the second SEC system. Then, we completed the analysis of the remaining compounds; the results are reported in Table 4. The reaction conditions used to prepare the analysed compounds are reported in Table 5.

**Figure 2:**
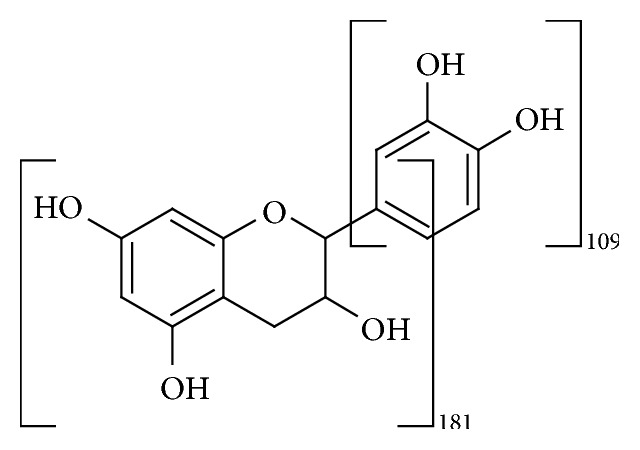
Fragmentation of epicatechin.

Maldi and SEC analyses confirmed the presence of two oligomers that lost the bicyclic part of epicatechin (Figure 2).

### 3.5. Effect of Epicatechin and of Oligomers from Epicatechin by HPI and by HRP on Melanoma Cell Line Proliferation

**Figure 3:**
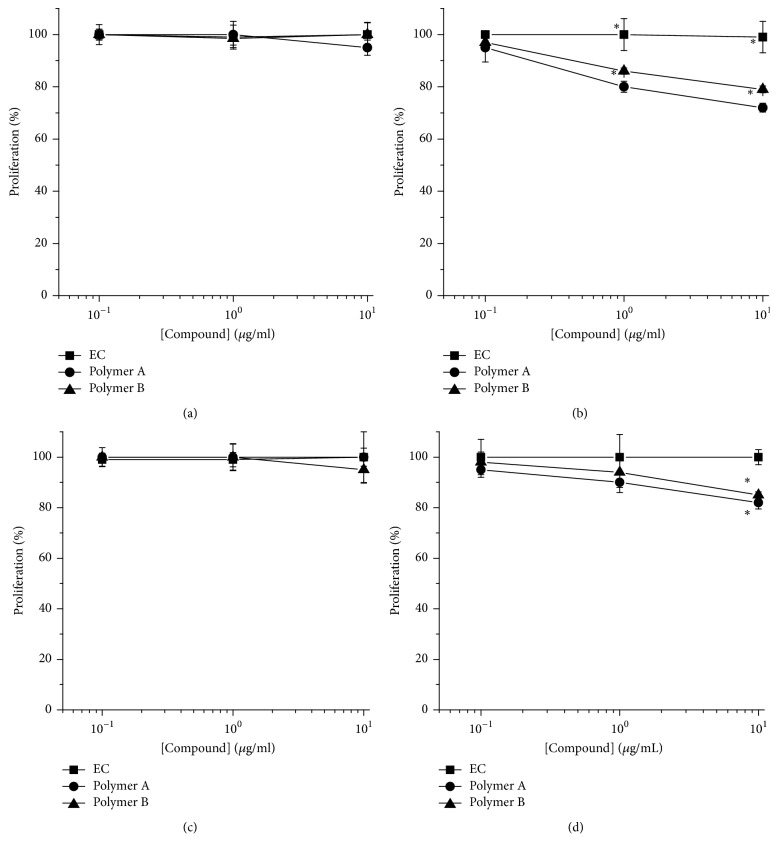
Oligomers effects on A375 (a and b) and WM266-4 (c and d) proliferation. Cells were treated with increasing concentrations (0.1–10 *μ*g/ml) of polymers A and B or EC for 3 (a and c) and 6 days (b and d). Cell proliferation was analysed by 3-(4,5-dimethylthiazol-2-yl)-2,5-diphenyl-tetrazolium bromide (MTT) assay and results were expressed as mean of percentage of the number of cells with respect to control cells. Data are shown as mean ± SEM. ^*∗*^*p* < 0.05.

Since catechins have been demonstrated to inhibit tumor cell line growth [2, 4, 30], the effect of epicatechin (EC) and one of the oligomers from epicatechin by HPI (called polymer A of 7 units of epicatechin) and one of the oligomers by HRP (called polymer B of 7 units of epicatechin) was first evaluated on melanoma cell line proliferation by MTT assay. As shown in Figure 3 the treatment (6 days) of WM266-4 cell line with 10 *μ*g/mL of polymer A or polymer B resulted in a slight but significant (*p* < 0.05) inhibition (−18% and −15 for polymer A and polymer B, resp.) of cell proliferation. Differently, the treatment (6 days) of A375 cell line induced a significant (*p* < 0.05) concentration dependent reduction of proliferation with a maximum of −28% and −21%, at 10 *μ*g/mL for polymer A and polymer B, respectively. In contrast, no effect was observed for EC on both cell lines at any tested concentrations and times.

### 3.6. Effect of EC, Polymer A, and Polymer B on Melanoma Cell Cycle

**Figure 4:**
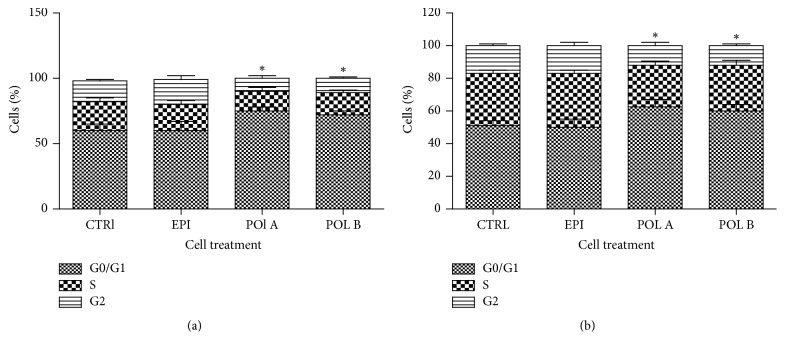
Oligomers effects on A375 (a) and WM266-4 (b) cell cycle. Cells were treated (10 *μ*g/ml) with polymers A and B or EC for 6 days and cell cycle was analysed by fluorescence-activated cell sorter (FACS). Results are expressed as percentage of the cell population in each phase. Data are shown as mean ± SEM (standard error of mean). ^*∗*^*p* < 0.05.

To further investigate the mechanism by which polymer A and polymer B affect melanoma cell line proliferation, the cell cycle was analysed. A375 or WM266-4 cells were treated with the compound and were shown to be active on both cell lines in the proliferation assay (10 *μ*g/mL) for 6 days and the distributions on cell cycle analysed by flow cytometry. As shown by PI staining, exposure to polymer A and polymer B (Figure 4) induced similar effects on both cell lines, an increase in the cell number in the G0/G1 phases (+22% and +17%, resp., for A375 cells; +25% and +20%, resp., for WM266-4 cells) accompanied by a reduction of cell number in S and G2 phases when compared with control cells, indicating an arrest in G0/G1 phases in cell cycle progression. Moreover a slight increase in debris was observed with both polymer A and polymer B. EC treatment did not induce any significant alteration in WM266-4 and A375 cells.

### 3.7. Effects of EC, Polymer A, and Polymer B on Cell Viability

**Figure 5:**
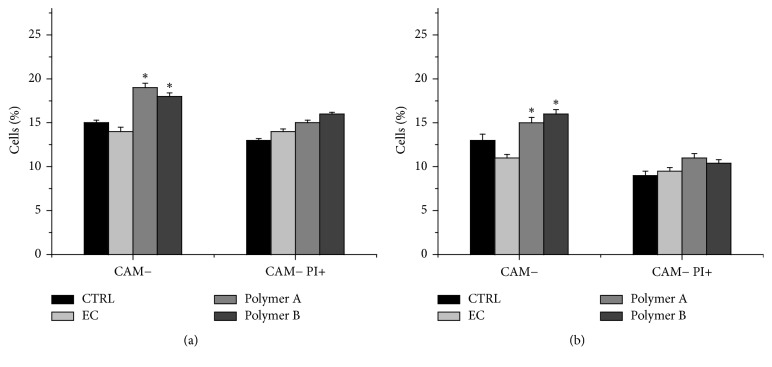
Oligomers effects on A375 (a) and WM266-4 (b) viability. Cells were treated with polymers A and B or EC (10 *μ*g/ml) for 6 days and cell death was analysed by fluorescence-activated cell sorter (FACS). Results are expressed as percentage of apoptotic and late apoptotic/necrotic cells in all samples. Data are shown as mean ± SEM. ^*∗*^*p* < 0.05. CAM− identifies apoptotic cells (Calcein-AMlow) and CAM− PI+ identifies late apoptotic/necrotic cells (Calcein-AMlow, propidium iodide treated).

To evaluate whether tested compounds exert also cytotoxic effects on tumor cells a CAM assay was performed. CAM labelled A375 and WM26-4 were treated (6 days) with 10 *μ*g/mL of each compound, counterstained with PI and analysed by FACS. As shown in Figure 5 both polymer A and polymer B induced an increase in CAM negative cells (apoptotic cells) (+26% and +20%, resp., for A375 cells; +15% and +23%, resp., for WM266-4 cells), and a slight increase in CAM^−^/PI^+^ cells (necrotic/late apoptotic cells). Conversely no significant cell death was observed in cell treated with EC.

## 4. Discussion

The interest towards enzymatic polymerization reactions catalyzed by peroxidases is growing, because they can be considered a valid alternative to chemical catalysis [5, 6, 12, 17]. Besides hydrolytic enzymes, oxidative enzymes represent a significant portion of these catalysts; in particular, fungal and plant oxidative enzymes are often used, while bacterial oxidative enzymes are still quite rare. However, enzymes from bacteria are easier to prepare because they are easily cultivated and many efficient procedures for their genetic manipulation have become standard in biomolecular studies. Consequently, the possibility of substituting enzymes of other origins with microbial alternatives is of great interest. Oxidative metal enzymes are widespread in bacteria. An issue in enzyme-mediated oxidative polymerizations is the number and variety of accepted substrates. Often, simple substrates (e.g., phenols) are easily transformed, while more complex ones (e.g., polyphenols) cannot be used.

*E. coli* HPI is a potential oxidative biocatalyst because it has both catalase and peroxidase activity [31, 32]. HPI is a metal based enzyme, containing Fe(II) in its catalytic site and its activity is connected to the oxidation state changes of iron. In the case of HPI it is also known that its catalase activity takes place at the iron site. Hydrogen peroxide is a small molecule that can easily penetrate the enzyme reaching the metal site. The issue becomes more complex when considering molecules of bigger size (as epicatechin) and growing polymers. In this paper, a recombinant HPI enzyme from* E. coli* was used in two different forms: the enzyme immobilized on the IMAC resin and the purified enzyme. All the preparations are active in test oxidative experiments, showing negligible differences. In contrast, the activity retention is different depending on the preparation. The enzyme immobilized on the resin is still fully active after two months, when it is conserved at 4°C. The free enzyme fast loses its activity at both 4°C and −20°C. This different behaviour can be explained considering the enzyme specific form. When it is blocked on the resin the enzyme resides inside the polymeric support; as a consequence, its 3D structure is stabilized and the activity is maintained for long time. Here, the temperature is less important. When in the free form, the enzyme is subjected to the solvent effect without any stabilization; thus, it tends to lose its native conformation showing an overall loss of activity. In conclusion, the enzymatic resin is both active and stable; in addition, it can be used several times.

In a previous work HPI was used in polymerization reactions of some aromatic substrates [16]. Results confirmed that the enzyme can act as a catalyst in oxidative polymerizations. Depending on the substrate the prepared oligomers had a specific length; however, the use of polyphenols as substrate was still to be tested. In addition, from the literature it is known that HRP can be used to prepare oligomers of catechins that can be interesting for their biological activity [4]. Everything considered, it is interesting to test the use of HPI for the preparation of (−)-epicatechin oligomers. Experiments with different combinations of conditions to obtain different epicatechin polymers were performed, and this was also done using HRP, to have a comparison. Results demonstrated that reactions performed with HRP or HPI completely consumed the substrate in line with literature data [4]. However, the oligomer length depends on several factors; the most important ones are the number of the started polymerization chains and their lifetime. Decreasing the substrate/enzyme ratio a smaller number of chains are activated and if their lifetime is long enough, the produced oligomer is longer. In contrast, because the polymerization is sustained by the oxidation state of the catalyst, decreasing the H_2_O_2_ concentration decreases the lifetime of the chain that therefore gives shorter oligomers (compare products of experiments P2 and P6) (Tables 4 and 5). The availability of the H_2_O_2_ is influenced by the competition of the two HPI activities: the catalase and the peroxidase ones. Finally, the insoluble product obtained in one experiment lost part of one epicatechin unit. This can be related to the mechanism of chain termination that can derive from a hydrogen loss or gain, or from a molecule part loss. When using the enzyme resin the product resembles that of the insoluble product from HRP; this seems to confirm that the chain termination is different in solution or in solid phase.

In conclusion of this part, it is possible to state that HPI in the immobilized form can be used to catalyze polyphenol polymerization; that it is a stable catalyst; and that it can be used several times without losing its activity. In comparison to HRP, obtained products are similar and HPI can be considered a valuable alternative.

The last part of the present study concerns the biological activity of the prepared oligomers.

The biological tests have been performed on two samples (one from HRP and one from HPI, called polymers A and B, resp.) chosen because they have the same length. Oligomer samples used in this study were purified before testing and their biological activity was investigated on the proliferation cell cycle. In both utilized cell lines, exposure to oligomers induced a reduction in tumor cell proliferation. The reduction in cell proliferation was primarily due to an arrest of the cell cycle in G0/G1 phase with a concurrent reduction in S phase. As shown by viability evaluation as a result of cell cycle arrest some cells undergo apoptosis. The ability of many catechins to inhibit tumor cell proliferation or exert cytotoxicity is well documented in many* in vitro* tumor models. As reported in literature catechins can differently affect tumor cell lines depending on their structure and on dose and time of treatment and type of intracellular trafficking in the cells [27, 33]. However, some authors suggest that also the degree of polymerization can affect catechin efficacy; in fact, Pierini et al. [34] showed that nongalloylated flavan-3-ols apple-derived monomer and dimers do not affect tumor cells proliferation and viability, while the oligomers and polymers are effective. Agarwal et al. [35] observed a correlation between the degree of polymerization and the antiproliferative activity on prostate cancer cell lines. We have not observed any effect on cell proliferation, viability, and cell cycle with epicatechin in our tumor models differently with respect to what was reported in literature on catechin as EGCG effects on human fibrosarcoma cells [33]. These results could depend on the tumor cell line selected; in fact literature analysis reveals that the epicatechin effects are dependent on selected tumor cells [2, 3]; another explanation could be the low concentrations utilized when compared with that which was shown to be active in other models [23, 30].

The overall results are consistent with others reported in the literature [4] where the same kinds of oligomers with a similar biological activity were obtained. The novelty of this work is that an alternative enzyme as HPI versus HRP can be used to prepare oligomers (A and B). Moreover, in this paper we demonstrated that the oligomeric forms of epicatechin are more effective than its monomer in the reduction of tumoral cell proliferation and that the reduction in cell proliferation was primarily due to an arrest of the cell cycle in G0/G1 phase with a concurrent reduction in S phase.

## 5. Conclusions

In conclusion, HPI enzyme can be considered a valuable alternative to vegetal derived enzymes in the preparation of (−)-epicatechin oligomers with a biological activity against tumor proliferation. Even if much work is still to be done, HPI can represent a good biocatalyst and epicatechin oligomers merit some attention as antitumoral agents.

## Figures and Tables

**Table 1:** Specific activity of HPI on resin on pyrogallol at 4°C.^a^

Time	Specific activity
1 d	2.0*∗*10^−1^ ± 2.2*∗*10^−2^
31 d	4.5*∗*10^−1^ ± 1.3*∗*10^−1^
41 d	5.4*∗*10^−1^ ± 5.6*∗*10^−2^
58 d	2.6*∗*10^−1^ ± 9.0*∗*10^−2^
121 d	3.2*∗*10^−1^ ± 4.7*∗*10^−2^

^a^ *μ*mol min^−1^ mg^−1^.

**Table 2:** Epicatechin consumed by immobilized HPI in different reaction conditions.

Reaction conditions	H_2_O_2_ (mM)	Enzyme (mg/mL)	Consumed epicatechin
Epicatechin 3.44 mM + EtOH 5%	15.5	0.00	No reaction
Epicatechin 0.69 mM + EtOH 20%	11.2	0.01	>90%
Epicatechin 3.44 mM + EtOH 5%	15.5	0.01	>90%
Epicatechin 3.44 mM + EtOH 30%	8.8	0.01	>90%
Epicatechin 17.2 mM + EtOH 30%	52.9	0.01	>25%

**Table 3:** Epicatechin consumed by purified HPI.

H_2_O_2_ additions	Consumed epicatechin
0 [0.0 mM]	0%
1 [3.9 mM]	31%
2 [7.8 mM]	31%
3 [11.7 mM]	33%
4 [15.6 mM]	48%
5 [19.5 mM]	48%
6 [23.4 mM]	50%
7 [27.3 mM]	50%
8 [31.2 mM]	57%
9 [35.1 mM]	57%

**Table 4:** MW of the obtained oligomers.

Compound	Retention time^a^	Measured MW^b^	Calculated MW^c^	Units^d^	Enzyme
epicatechin	10.17	331^e^		1	
P1	6.80	2326	2038	7	HRP
P2	7.33		1520	6^f^	HPI
P3	7.32	1724	1529	6^f^	HRP
P4	6.94		1885	7^f^	HRP
P5	6.95		1875	7^f^	HPI
P6	6.89		1937	7	HPI

^a^Measured using SEC-2 system. ^b^Calculated using SEC-1 system. ^c^From the correlation line. ^d^Number of epicatechin units. ^e^Corrected weight using Maldi/SEC ratio. ^f^From the compound that lost the bicyclic part.

**Table 5:** Conditions of reaction utilized for the obtained oligomers.

Compound	Conc. EPI	Enz.	EtOH	mL^a^
P1	3.44 mM	1 mg HRP	20%	20
P2^b^	3.44 mM	0.01 mg HPI	5%	1
P3	3.44 mM	0.01 mg HRP	5%	1
P4	3.44 mM	0.01 mg HRP	5%	20
P5	0.69 mM	0.05 mg HPI	20%	2.5
P6^c^	3.44 mM	0.01 mg HPI	5%	1

^a^Reaction volume. ^b^3 mL of H_2_O_2_ added. ^c^42 mL of H_2_O_2_ added.

## Data Availability

The data used to support the findings of this study are available from the corresponding author upon request.
